# Unique model of chronic hypoxia in fetal lambs demonstrates abnormal contrast-enhanced ultrasound brain perfusion

**DOI:** 10.1038/s41390-024-03206-3

**Published:** 2024-06-07

**Authors:** Divyansh Agarwal, Mallory L. Hunt, Anush Sridharan, Abby C. Larson, Jack Rychik, Daniel J. Licht, Marcus G. Davey, Alan W. Flake, J. William Gaynor, Ryne A. Didier

**Affiliations:** 1 Perelman School of Medicine, University of Philadelphia, Philadelphia, PA USA; 2 Department of Surgery, Massachusetts General Hospital, Harvard Medical School, Boston, MA USA; 3 Department of Surgery, Children’s Hospital of Philadelphia, Philadelphia, PA USA; 4 Center for Fetal Research, Children’s Hospital of Philadelphia, Philadelphia, PA USA; 5 Department of Radiology, Children’s Hospital of Philadelphia, Philadelphia, PA USA; 6 Department of Cardiology, Children’s Hospital of Philadelphia, Philadelphia, PA USA; 7 Department of Neurology, Children’s Hospital of Philadelphia, Philadelphia, PA USA

## Abstract

**Background:**

Children with congenital heart disease (CHD) demonstrate long-term neurodevelopmental impairments. We investigated contrast-enhanced ultrasound (CEUS) cerebral perfusion in a fetal animal model exposed to sub-physiologic oxygen at equivalent levels observed in human fetuses with CHD.

**Methods:**

Fifteen fetal lambs [hypoxic animals (*n* = 9) and normoxic controls (*n* = 6)] maintained in an extrauterine environment underwent periodic brain CEUS. Perfusion parameters including microvascular flow velocity (MFV), transit time, and microvascular blood flow (MBF) were extrapolated from a standardized plane; regions of interest (ROI) included whole brain, central/thalami, and peripheral parenchymal analyses. Daily echocardiographic parameters and middle cerebral artery (MCA) pulsatility indices (PIs) were obtained.

**Results:**

Hypoxic lambs demonstrated decreased MFV, increased transit time, and decreased MBF (*p* = 0.026, *p* = 0.016, and *p* < 0.001, respectively) by whole brain analyses. MFV and transit time were relatively preserved in the central/thalami (*p* = 0.11, *p* = 0.08, *p* = 0.012, respectively) with differences in the peripheral parenchyma (all *p* < 0.001). In general, cardiac variables did not correlate with cerebral CEUS perfusion parameters. Hypoxic animals demonstrated decreased MCA PI compared to controls (0.65 vs. 0.78, respectively; *p* = 0.027).

**Conclusion:**

Aberrations in CEUS perfusion parameters suggest that in environments of prolonged hypoxia, there are regional microvascular differences incompletely characterized by MCA interrogation offering insights into fetal conditions which may contribute to patient outcomes.

**Impact:**

- This work utilizes CEUS to study cerebral microvascular perfusion in a unique fetal animal model subjected to chronic hypoxic conditions equal to fetuses with congenital heart disease. CEUS demonstrates altered parameters with regional differences that are incompletely characterized by MCA Doppler values.
- These findings show that routine MCA Doppler interrogation may be inadequate in assessing microvascular perfusion differences.
- To our knowledge, this study is the first to utilize CEUS to assess microvascular perfusion in this model.
- The results offer insight into underlying conditions and physiological changes which may contribute to known neurodevelopmental impairments in those with congenital heart disease.

## Introduction

Infants and children with congenital heart disease (CHD) demonstrate long-term neurologic sequelae, including impairments in cognition, language, and executive function.^1–4^ Intraoperative and postnatal factors do not fully explain variation in developmental outcomes among individuals with CHD, suggesting that patient-specific and prenatal factors influence adverse long-term neurodevelopmental processes.^5,6^ These effects are hypothesized to occur, in part, from a relative state of chronic hypoxia, which influences cardiovascular physiology and neurologic development and maturation.^7–11^ In utero evaluation of cerebrovascular physiology has been limited to grayscale and colour Doppler ultrasound (US) and magnetic resonance imaging (MRI) with findings including small brain biometric measurements and altered middle cerebral artery Doppler measurements.^12–16^ However, these modalities have limitations; for example, US is unable to measure functional data and motion artefacts, cost, and accessibility obstacles tend to occur with MRI. Furthermore, middle cerebral artery (MCA) flow patterns are most commonly used to assess cerebral blood flow but the correlation of this information with microvascular or regional blood flow is not known. Thus, the need for advanced imaging technologies to overcome these limitations has become paramount.

Contrast-enhanced ultrasound (CEUS) is a validated method of examining microvascular cerebral perfusion and, among many applications, clinical CEUS of the pediatric brain has been used to evaluate the neonatal hypoxic–ischemic injury, brain tumour enhancement characteristics for surgical planning, and intraoperative monitoring of brain perfusion.^17–21^ Much of the existing literature has focused on CEUS as a sensitive modality in evaluating acute ischemia,^22–24^ but to our knowledge, little is known about the imaging evaluation in the fetus or of the chronic hypoxic state as with CHD.

Recent developments in support devices to simulate the maternal womb, including the EXTrauterine Environment for Neonatal Development (EXTEND) system, have allowed the simulation and investigation of a variety of prenatal disease processes such as CHD.^25^ One model has been developed to mimic the cerebrovascular effects of CHD by creating a state of chronic hypoxia in fetal lambs, which has been shown to result in abnormal neurodevelopment similar to that seen in children with CHD.^26,27^ This model provides a unique research platform to investigate emerging imaging technologies such as CEUS to better understand the in utero physiology that may lead to neurologic sequelae. Prior investigations have demonstrated the feasibility and safety of CEUS in the EXTEND system.^28,29^ The purpose of this study was to evaluate CEUS cerebral perfusion parameters in a fetal model of altered oxygenation similar to that of a fetus with CHD.

## Methods

### Surgical procedure

In accordance with IACUC-approved protocols, 15 ewes underwent general anaesthesia and surgical hysterotomy was performed. Fifteen fetal lambs at 106–111 days gestational age (7 female and 8 male) were transferred from placental support to the EXTEND system, per published technique, where term is approximately 145 days gestational age.^25^ Nine lambs were randomized to the hypoxic group and after 24 h on the EXTEND system were subjected to sub-physiologic oxygen levels (target of 14–16 cmL/kg/min; 15.6 ± 1.0 mL/kg/min), similar to levels in a fetus with CHD for the remainder of the experimental period.^26^ The remaining six control lambs were exposed to physiologic oxygen levels (target of > 20 mL/kg/min; 21.6 ± 2.0 mL/kg/min). All animals were maintained on circuit for up to 21 days per clinical protocol. Continuous hemodynamic monitoring including circuit flow and oxygen delivery measurements were recorded. As per published techniques, carbon dioxide levels were maintained via sweep gas at the oxygenator at 35–45 mmHg and all animals were supported with similar caloric delivery.^25–27^

### Imaging protocol

**Fig. 1:**
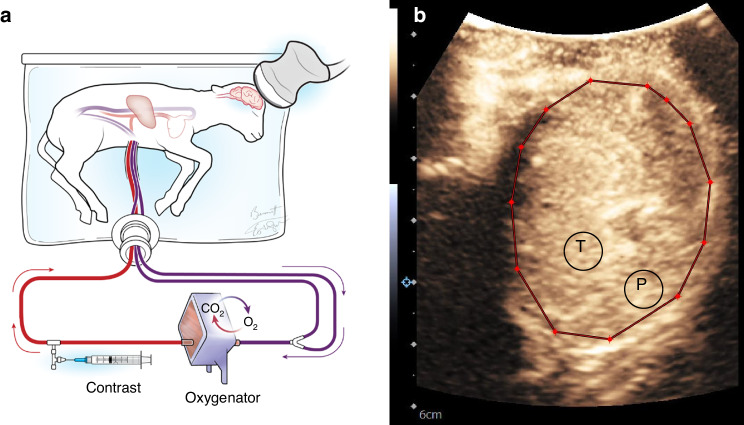
Experimental set-up for contrast-enhanced ultrasound (CEUS) of the brain. **a** Illustration depicting the fetal lamb within the EXTrauterine Environment for Neonatal Development (EXTEND) undergoing CEUS of the brain with US contrast administered into the fetal circulation (syringe) distal to the oxygenator with imaging performed of the fetal brain. **b** Representative transaxial image through the fetal lamb brain at peak enhancement during CEUS examination with regions of interest (ROI) including whole brain (red circle), central/thalamic region (T), and peripheral parenchyma (P).

**Fig. 2:**
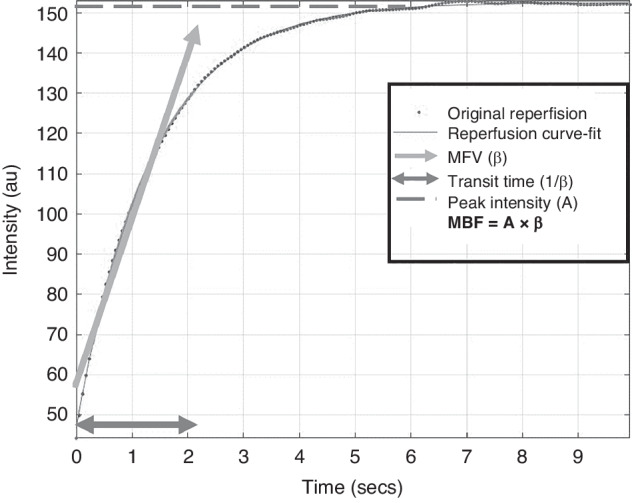
Time–intensity curve. Time–intensity curve extrapolated from flash replenishment cine acquisition from contrast-enhanced ultrasound examination depicting ultrasound contrast intensity over time (dotted curve) with overlying fitted curve (smooth curve) from which cerebral perfusion parameters are extrapolated including microvascular flow velocity (MFV), transit time, and microvascular blood flow (MBF). au arbitrary unit, secs seconds.

A Siemens Accuson Sequoia US system (Siemens Medical Solutions, Inc. Malvern, Pennsylvania) and 9C3 curved array transducer optimized for contrast visualization was utilized to perform the CEUS examinations at various time points depending on lamb stability, sonographer availability, and time on the circuit. Images were obtained in the transaxial plane at the thinnest portion of the lamb calvarium with the field of view to include the frontoparietal brain parenchyma and basal ganglia/thalami for localization (Fig. 1a). Axial and lateral resolution of this transducer is 0.56 mm and 0.96 mm, respectively. Definity (Lantheus Medical Imaging, North Billerica, Massachusetts) US contrast was prepared per manufacturer guidelines and administered by infusion into the umbilical vein distal to the circuit oxygenator at 120 mL/h. After achieving a steady state, multiple flash replenishment sequences were performed which consisted of a 3-sec microbubble destruction sequence designed to destroy all microbubbles in the field of view followed by 15 sec of imaging and saved as a cine clip. Cine clips were then analyzed using MatLab (The Mathworks Inc., Natick, Massachusetts) with ROIs to include a) the whole brain and b) 5-mm diameter circular region of the basal ganglia/thalami and c) 5-mm diameter circular region in the peripheral cerebral parenchyma which includes predominately white matter (Fig. 1b) with attempts to avoid the overlying cortex (<1 mm thick) noting that this could not be visually confirmed as the images were optimized for contrast visualization which decreases conspicuity of the grey–white junction. Time–intensity curves were generated and microvascular perfusion parameters including microvascular flow velocity (MFV), transit time, and microvascular blood flow (MBF) were extrapolated (Fig. 2). Recorded parameters are based on existing literature validating their use in CEUS image analysis.^21,30–32^ MFV is a measure of the speed at which the red blood cell enters the cerebral microvascular circulation in relation to peak intensity and quantifies regional blood flow velocity. Transit time represents the time the red blood cell spends within the capillary bed crossing from arterial to venous systems. MBF is a surrogate measurement of cerebral blood flow and is related to the estimated blood volume (peak intensity of US contrast in an area of tissue following a flash replenishment sequence) divided by the transit time. This parameter has been shown to correlate with cerebral blood flow measured by radiolabeled microspheres and is routinely reported in CEUS literature.^21^ Measurements were excluded if the curve fit was unreliable, as determined by *R*^2^ < 0.70.

### Cardiac output and middle cerebral artery Doppler data

Daily echocardiography was performed to determine volumetric cardiac blood flow parameters across the aorta and pulmonary artery providing for left ventricular cardiac output (LVCO), right ventricular cardiac output (RVCO), and combined cardiac output (CCO); and from these parameters, the shunt fraction and the ratio of LVCO to RVCO (LVCO:RVCO) were calculated. MCA Doppler evaluation was performed immediately prior to the CEUS examination and pulsatility indices (PIs) were produced automatically by the US system software and the PI was recorded. If multiple Doppler waveforms were obtained, the PI from the clearest waveform with the least motion and technical artefact was used for analyses.

#### Statistical analyses

Differences in CEUS cerebral perfusion parameters were compared between groups using the statistical software R version 4.0.3 (R Core Team, Vienna, Austria), including the whole brain, central/thalamic region, and peripheral brain region. The absolute difference and ratio between central/thalamic region and peripheral brain region perfusion parameters were compared with the nonparametric Mann–Whitney *U* tests with continuity correction. These tests were also performed following stratification by time on circuit and duration of hypoxemia where studies performed at ≤4 days were deemed “early,” those ≥9 days were considered “late,” and those in between 5 and 8 days were considered “intermediate.” Multivariate crossmatch statistic was computed to compare MFV to transit time relationship in each group.^30^ Cardiac parameters and MCA PIs were compared between groups using the Mann–Whitney *U* test. Correlations between cardiac and brain CEUS parameters were investigated using Spearman’s rho (rank-order correlation). All p-values were adjusted for multiple hypothesis testing using the Benjamini–Hochberg correction to account for a false discovery rate. Statistical significance was established at an adjusted *p* value ≤ 0.05.

## Results

**Fig. 3:**
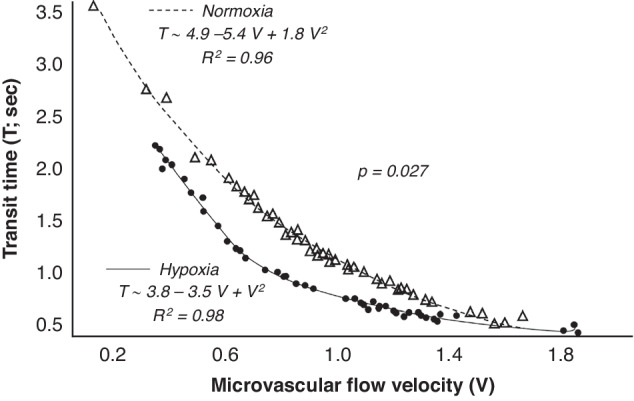
Multivariate crossmatch results evaluating relationship between transit time and microvascular flow velocity (MFV) Hypoxic animals (•) demonstrated smaller increases in transit time with decreasing MFV when compared to normoxic controls (∆). Regression curve fit equations and coefficients of determination (*R*^2^) are listed and lines of best fit overly the plotted data points for each group where the variable *V* represents the MFV.

Each lamb underwent 1–3 CEUS examinations separated by at least 24 h at a mean of 6.3 days on circuit (range 2–13). When including all examinations, in whole brain analyses, fetal lambs in the hypoxia group demonstrated decreased MFV, increased transit time, and overall decreased MBF when compared to normoxic controls (Table 1). Evaluation of results from the smaller ROI analyses demonstrated that the decreased MFV, increased transit time, and decreased MBF were significant in the peripheral cerebral parenchyma (Table 1). Decreased MBF was also demonstrated in the central thalami but there were no significant differences in MFV or transit time in this smaller ROI (Table 1). When evaluating the difference in perfusion metrics between the central thalami and peripheral parenchyma, the absolute difference in MFV was statistically significant (*p* = 0.002) while there was no difference in transit time or MBF (*p* = 0.082 and *p* = 0.095, respectively). However, there was a significant difference in the ratio of thalamic and peripheral parenchymal parameters for MFV, transit time, and MBF (*p* = 0.001, *p* = 0.003, and *p* = 0.001, respectively). We also observed a significant differential relationship between MFV and transit time in the hypoxic animals compared to the normoxic controls. Hypoxic animals demonstrated smaller increases in transit time with incremental decreases in MFV (crossmatch test *p* = 0.027) (Fig. 3). The choice of a parabolic function here to model the empiric relationship between the two variables more accurately captures the nonlinear behaviour of the variables, and allows for a distribution-free comparison of the differences between the two groups.

**Table 1:** Comparison of contrast-enhanced ultrasound perfusion parameters between groups in the whole brain, central/thalamic region, and peripheral parenchyma (median ± interquartile range).

	Hypoxic	Normoxic	*p* value
**MFV** **(au/s)**			
Whole brain	0.78 ± 0.36	1.1 ± 0.42	0.026*
Central/thalamic	1.32 ± 1.01	1.47 ± 0.88	0.11
Peripheral parenchyma	0.59 ± 0.51	0.84 ± 0.69	<0.001*
**Transit time (s)**			
Whole brain	0.99 ± 0.46	0.63 ± 0.22	0.016*
Central/thalamic	3.65 ± 1.47	3.17 ± 1.33	0.08
Peripheral parenchyma	3.84 ± 1.99	1.87 ± 0.92	<0.001*
**MBF** **(au**^**2**^**/s)**			
Whole brain	109 ± 84	181 ± 57	<0.001*
Central/thalamic	211 ± 136	242 ± 106	0.012*
Peripheral parenchyma	68 ± 27	102 ± 45	<0.001*

*MFV* microvascular flow velocity, *au* arbitrary units, *s* second, *MBF* microvascular blood flow.

* Denotes statistically significant results.

**Fig. 4:**
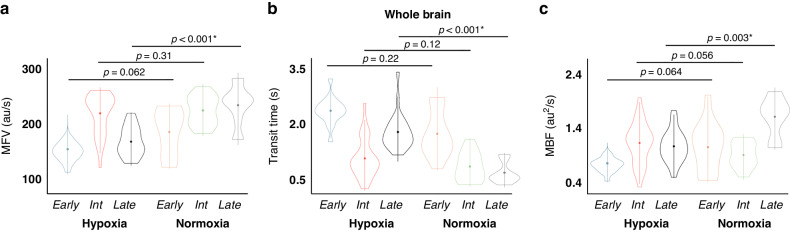
Comparison of perfusion parameters in the whole brain by days on the EXTEND circuit where early, intermediate, and late examinations are ≤ 4, 5–8, and ≥ 9 days on circuit, respectively. Significant differences in **a** Microvascular flow velocity (MFV) **b** transit time and **c** microvascular blood flow (MBF) were demonstrated at later time points (≥9 days on circuit) with no statistically significant differences observed between hypoxic and control animals at earlier or intermediate time points.

**Fig. 5:**
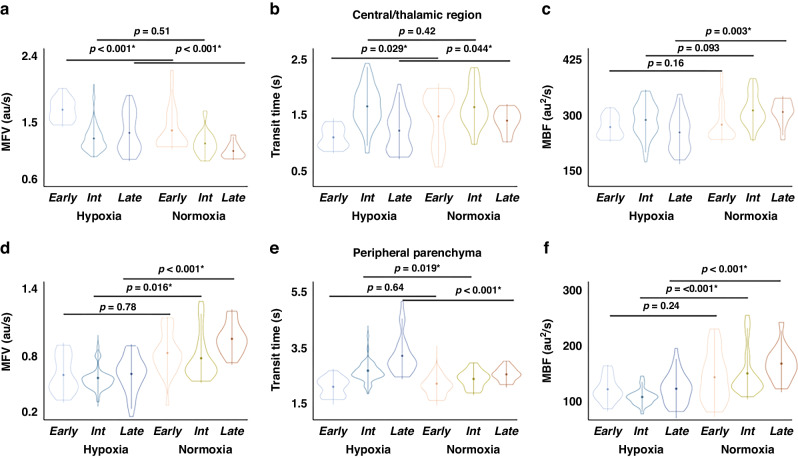
Comparison of perfusion parameters in the central/thalamic (top) and peripheral parenchymal (bottom) regions by days on the EXTEND circuit where early, intermediate, and late examinations are ≤ 4, 5–8, and ≥ 9 days on circuit, respectively. Centrally, greater differences in **a** Microvascular flow velocity (MFV) and **b** transit time are observed in early (8 data points) and late examinations (6 data points) but not in the intermediate group (14 data points). Differences in **c** microvascular blood flow (MBF) were only observed in the late group. In the peripheral parenchyma, significant differences in all perfusion parameters including **d** MFV, **e** transit time, and **f** MBF are observed with both intermediate and late experiments but not the early group. Significant results are denoted by an asterisk (*); MFV microvascular flow velocity, au arbitrary unit, s seconds, int intermediate, MBF microvascular blood flow.

Differences in global perfusion parameters were most apparent at later time points in the experiment (≥9 days on circuit) with no statistically significant differences observed between hypoxic and control animals at earlier or intermediate time points (≤4 days and 5–8 days on circuit, respectively, Fig. 4). Figure 5 demonstrates the differences in CEUS perfusion parameters in the central/thalamic and peripheral parenchymal regions for all groups based on age. In the central/thalamic regions, differences in MFV and transit time were observed in both the early and late groups, but not the intermediate group. Cerebral MBF was significantly decreased in the thalami only in the animals ≥ 9 days on circuit. Significant differences in CEUS perfusion parameters were observed in the peripheral parenchyma for both intermediate and late groups, but not in those maintained on circuit for ≤ 4 days.

**Fig. 6:**
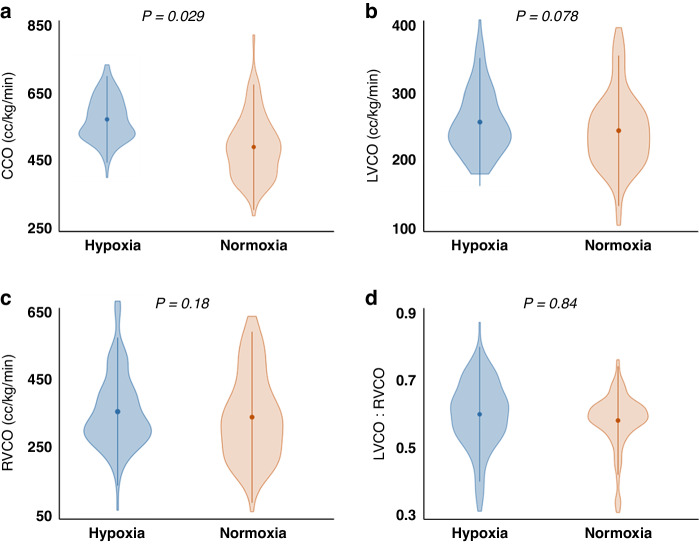
Comparison of cardiac parameters of hypoxic and normoxic animals. Compared to normoxic controls, hypoxic animals demonstrated **a** significantly increased combined cardiac output (CCO) and **b** a trend toward increased left ventricular cardiac output (LVCO) but there were no significant differences between **c** right ventricular cardiac output (RVCO) and **d** LVCO:RVCO ratios.

Hypoxic animals demonstrated statistically significant increased CCO as well as a trend toward increased LVCO when compared to normoxic animals but there were no statistically meaningful differences between RVCO and LVCO:RVCO between the two groups (Fig. 6). When compared to normoxic control animals, hypoxic animals showed an expected decrease in median circuit flows (221 mL/kg/min vs. 278 mL/kg/min; *p* < 0.001) and median shunt fraction (0.29 vs. 0.35; *p* < 0.001), as lower circuit flows and shunting to the oxygenator were required to achieve a lower target oxygen delivery compared with normoxic counterparts. By linear regression analysis, there was no significant correlation between most brain perfusion and cardiac parameters (Table 2). Hypoxic animals also demonstrated significantly decreased MCA PI when compared to controls (0.65 vs. 0.78, respectively; *p* = 0.027).

**Table 2:** Correlations between cardiac physiologic and contrast-enhanced ultrasound perfusion parameters in the whole brain, central/thalami, and peripheral parenchyma by group.

		CCO (cc/kg/min)				LVCO (cc/kg/min)				RVCO (cc/kg/min)				LVCO:RVCO			
		Hypoxic		Normoxic		Hypoxic		Normoxic		Hypoxic		Normoxic		Hypoxic		Normoxic	
		*r*	*p*	*r*	*p*	*r*	*p*	*r*	*p*	*r*	*p*	*r*	*p*	*r*	*p*	*r*	*p*
**Whole brain**	MFV (au/s)	−0.16	0.55	0.01	1	−0.13	0.63	0.01	0.97	0.03	0.89	0.05	0.87	−0.29	0.23	0.14	0.67
	Transit time (s)	0.15	0.56	−0.02	0.56	0.15	0.55	−0.04	0.91	0.04	0.81	−0.06	0.85	0.28	0.27	−0.16	0.61
	MBF (au^2^/s)	−0.16	0.54	0.13	0.68	−0.09	0.72	0.13	0.68	−0.06	0.81	0.11	0.74	−0.24	0.36	0.18	0.59
**Central/thalamic**	MFV (au/s)	−0.27	0.37	0.54	0.08	−0.06	0.83	−0.58	0.05	−0.14	0.63	0.49	0.11	−0.11	0.71	0.27	0.41
	Transit time (s)	0.17	0.56	−0.51	0.1	0.02	1	−0.56	0.06	0.03	0.94	−0.48	0.12	0.14	0.62	−0.29	0.38
	MBF (au^2^/s)	−0.12	0.68	0.48	0.12	0.07	0.81	0.51	0.1	−0.13	0.68	−0.39	0.21	0.041	0.89	0.32	0.29
**Peripheral parenchyma**	MFV (au/s)	−0.33	0.19	0.42	0.16	−0.05	0.86	0.42	0.18	−0.51	0.04*	0.37	0.23	−0.04	0.91	0.21	0.51
	Transit time (s)	0.25	0.32	−0.49	0.11	−0.01	0.99	−0.47	0.13	0.37	0.14	−0.44	0.16	0.19	0.52	−0.16	0.62
	MBF (au^2^/s)	−0.28	0.27	0.46	0.12	0.037	0.89	0.39	0.21	−0.48	0.06	0.37	0.22	−0.11	0.75	0.13	0.58

*CCO* combined cardiac output, *LVCO* left ventricular cardiac output, *RVCO* right ventricular cardiac output, *cc* cubic centimeter, *kg* kilogram, *min* minute, *r* Spearman’s rho, *p* *p* value adjusted, *MFV* microvascular flow velocity, *au* arbitrary units, *MBF* microvascular blood flow, *s* second

* Denotes statistically significant results.

## Discussion

Fetal lambs subjected to chronic hypoxic conditions as a model for CHD demonstrated altered parenchymal perfusion parameters by brain CEUS, including decreased MFV, increased transit time, and overall decreased brain MBF. The changes in MFV and transit time were relatively preserved in the central/thalamic regions compared to the peripheral parenchyma suggesting preservation of these parameters to the most primitive components of the brain required for survival at a consequence to the regions of the brain involved in higher executive functions. This is consistent with our understanding that the preservation of central blood flow is critical for neuronal differentiation and migration, while the metabolic demand of the mature peripheral neuron is less.^33^

In addition to these general aberrations in CEUS perfusion parameters, we demonstrated varying degrees of abnormality depending on length of time on circuit. For example, differences in global perfusion parameters were most apparent after chronic exposure to subphysiologic oxygen levels suggesting that the general observations reported in this study are predominately reflective of adaptations to chronic rather than acute or subacute hypoxic conditions. Furthermore, significant differences in MBF and transit time in the central/thalamic regions were observed in both early and late stages of evaluation suggesting responses in these parameters to both acute and chronic hypoxic conditions centrally. Conversely, changes in the peripheral parenchyma were not observed early in exposure to sub-physiologic oxygen levels suggesting that acute vascular compensatory mechanisms, which were eventually replaced by other adaptations such as decreasing utilization, led to changes in brain perfusion after approximately 1 week on circuit. In addition to these findings, hypoxic animals also demonstrated altered relationships between transit time and MFV when compared to normoxic controls, further supporting the hypothesis that these animals utilized and optimized compensatory mechanisms in response to relative hypoxia. These animals demonstrated significantly increased CCO and a trend toward increased LVCO but otherwise, there were no unexpected significant correlations between cardiac and CEUS perfusion parameters which suggests that cerebral compensatory mechanisms are independent of systemic changes but given the differences observed related to length of exposure to hypoxic conditions, our findings suggest that cerebral compensation is dynamic but inadequate in preserving MBF. Ultimately, MCA Doppler evaluation of global brain perfusion alone may be inadequate in characterizing the complex regional blood flow changes that occur in this model, as demonstrated in the abnormal CEUS perfusion parameters, and may be occurring in fetuses with congenital heart disease. Previous work in this animal model has been shown to reproduce many of the neuropathological findings in children with CHD including reduced neuronal density, increased white matter capillary density/vascularity, and decreased cortical myelin integrity suggesting impaired myelination.^26^ Therefore, this altered brain maturation in children with CHD and this animal model may be related to regional microvascular changes observed in this study and ineffectively characterized by routinely utilized large vessel ultrasound metrics such as MCA PI.

Aberrant CEUS brain perfusion parameters may shed light on the underlying mechanisms of neurological impairment demonstrated in children with a history of repaired congenital heart disease. The increased CCO observed in hypoxic animals suggests that there are intrinsic cardiac compensatory mechanisms to account for decreased oxygen levels. However, based on our results demonstrating no linear correlation between cardiac and brain perfusion parameters, these cardiovascular changes occurring at a macrovascular level may be inadequate in preserving regional microvascular cerebral blood flow. The decreased MFV was observed despite the increased CCO. As blood flow velocity is inversely proportional to the cross-sectional area of the vessels, this finding may be related to large vessel vasodilation which is supported by our finding of decreased MCA PIs in hypoxic animals compared to controls and suggests that this physiologic response to hypoxic conditions is inadequate to meet all regional demands. Decreased MCA PI, which reflects decreased vascular impedance, is a mechanism to increase cerebral blood flow, but we consistently demonstrated decreased MBF suggesting that decreased MCA PI does not assure increased regional blood flow and variability in capacity to provide for increased blood flow when impedance drops may lead to variability in perfusion. Furthermore, our finding of decreased MFV persisted in the smaller ROI analyses which excludes large vessels from the field of view, and therefore, these aberrations are favoured to reflect microvascular changes. The increased transit time, or greater time the red blood cell spends in the capillary bed, suggests intrinsic compensation to allow for greater oxygen extraction. This may be explained by altered capillary bed surface area via vasodilation or structural changes related to vascular density; our finding of greater aberrations later in exposure to sub-physiologic oxygen levels suggest chronic processes such as vascular remodeling rather than acute changes in vascular diameter and may correlate with previous experiments in this animal model demonstrating increased white matter capillary density/vascularity.^26^ in hypoxic animals when compared to controls. However, the lack of linear correlation between cardiac and brain perfusion parameters suggests that there was at least some preservation of cerebral autoregulation despite prolonged hypoxia.

Recent feasibility studies have utilized CEUS in infants undergoing surgical repair of CHD with and without cardiopulmonary bypass and have demonstrated altered cerebral perfusion as demonstrated by significant differences in rise time and time to peak over the course of the surgical intervention.^19^ Prior studies have investigated in utero MCA Doppler changes in fetuses with CHD demonstrating decreased cerebrovascular resistance with variation observed depending on the type and severity of the cardiac lesion which is concordant with our findings of increased transit time and decreased MCA PIs.^34–37^ However, our study is the first to utilize CEUS in evaluating prenatal brain perfusion in a model of CHD and the first to demonstrate regional differences in perfusion parameters in addition to large vessel changes. Given the gap in scientific knowledge regarding the underlying prenatal mechanisms contributing to neurodevelopmental impairments in these patients, the results from our current work offer a unique pathophysiological insight into the in utero processes that may contribute to altered cerebral development and subsequent neurological differences. Furthermore, our finding of decreased MCA PI while simultaneously demonstrating decreased global cerebral MBF suggests that routinely employed large vessel assessment may be inadequate in understanding what is occurring at the microvascular level. It is conceivable that the vasodilation and brain-sparing effects observed with upstream macrovascular evaluation may not overcome changes to the microvascular structures which lead to altered microvascular perfusion parameters demonstrated by CEUS.

Despite the promising application of CEUS to uncover the underlying neurovascular perturbations in CHD, our study has several limitations. For one, although the EXTEND model has been substantiated, it is plausible that the clinical laboratory findings in an animal model may not be applicable to human fetuses. Yet, given the lack of prenatal brain perfusion data in babies with CHD, our study offers the first assessment of in utero cerebral blood flow parameters in this model of chronic hypoxemia as a surrogate for CHD. Future studies investigating CEUS brain perfusion in humans will be imperative to corroborate our initial findings. Secondly, the relatively small sample size limits further statistical analyses and generalization of findings to a larger population. Third, given that the measurements of cardiac function were obtained daily at an independent time, and not in conjunction with the CEUS examinations, the correlation analyses between these parameters may be temporally limited. As US contrast agents interfere with echocardiographic assessment of the heart and vasculature, with potential biomechanical and cavitation implications, simultaneous evaluation of cardiac and brain perfusion parameters is not possible. Fourth, we acknowledge that the MBF parameter relies on peak intensity, or the concentration of microbubbles within the cerebral tissue, and may be affected by various factors including US beam attenuation, variations in positioning, and intrinsic animal differences. We aimed to control for these factors by employing an infusion dosing scheme which should provide a constant microbubble concentration, single sonographer acquisition, standardized imaging plane, and sequential triplicate acquisition. Furthermore, MBF is a rate-based parameter relying on the transit time which is known to be independent of microbubble concentration, is correlated with MBF as measured by radiolabeled microbubbles, and is routinely reported in the CEUS literature.^21,38^ Lastly, lack of robust histopathologic analyses limits the interpretation of the CEUS findings and these will be the focus of future studies.

To summarize, CEUS cerebral perfusion parameters are altered in this model of CHD, as exhibited through an animal model in the EXTEND system. The altered perfusion is most conspicuous in the peripheral parenchyma when compared to the central/thalamic regions and more pronounced at greater/chronic exposure to sub-physiologic oxygen levels. This shunting of cerebral blood flow to central brain structures may result in the observed changes in cortical and white matter development seen in both lambs raised in hypoxic environments and our CHD newborns. Our findings suggest that regional microvascular changes in response to the chronic hypoxic environment are incompletely characterized by upstream large vessel vasodilation observed by decreased MCA PIs. These aberrations in brain perfusion offer insights into the underlying in utero conditions which may contribute to known neurodevelopmental impairments in those with CHD.

The datasets generated during and/or analyzed during the current study are available from the corresponding author on reasonable request.
